# What Are Tyrotoxicon and Ptomaines?

**Published:** 1889-11

**Authors:** 


### WHAT ARE TYROTOXICON AND PTOMAINES?

Dr. W. T. Belfield quite recently discussed this subject with a representative of the press. His study of the matter enabled him to so clearly explain the cause of ptomaines and tyrotoxicon that we here publish some extracts from his remarks :

"Ptomaines," repeated the doctor, "are the products of micro-organisms. Let me first tell you that I referred to ice cream because the particular micro organic poison—tyrotoxicon—found in ice cream has been more carefully analyzed, studied and proved than any other of the infinite tiny fungoid growths. It is scarcely more than twenty years since bacteria were discovered, and not much more than half that period since their poisonous product was made known to the world. When you know that there are infinite forms of minute fungoid growths, and that each form has its specific product, you will understand that in so brief a time it is scarcely possible to have classified, arranged and studied the specific effects of their products in the system. With the poison of the ice cream, tyrotoxicon, however, it is different. That has been isolated and studied. It has been fed to animals and the poisonous properties preserved. Much of the knowledge which obtains concerning other forms has been secured by comparison with the ice cream poison."

"Suppose we get, first, a knowledge of what bacteria are?" suggested the reporter.

"Very well," assented the microscopist, whose researches had been profound. "You understand what a fungus is—a cellular, colorless parasite. Lacking the green of the flowering plants, it must go to some object for support. Bacteria are fungi, just as are the toadstools of field and wood—as are the delicious mushroom which the caterer serves with the juicy porterhouse steak. Now, bacteria are very minute fungi, most of them invisible to the unaided eye, and they flourish only inside animals. Note this fact : While all animate things cling to life, the lower forms cling with singular tenacity to it. Hence, I say the bacteria flourish only in animal bodies. They hold their being, however, in varied temperatures, springing into activity as the temperature rises above ninety degrees F. With the 'flowering' fungi and the 'budding' fungi, these forms being larger and their forms not having been studied, we come to the bacteria, which, without exception, perpetuate their species by inside budding. They are divided into three classes—the micrococcus, or globular in shape; the bacillus, rod-shaped and the spirillum, or spiral shaped."

"What diseases do these various forms develop?"

"Let me see. The results of the product of the first, micrococcus, are seen in the various forms of blood-poisoning—pyæmia, septicemia, &c. Remittent fever reveals the product of the third or the spirillum. Familiar forms of the bacillic products are consumption and splenic fever in cattle. Incidentally this disease made sad havoc with cattle until Pasteur successfully vaccinated against it."

"How do you say they bud, these several forms?"

"Within. Small buds from inside these plants, and at the fullness of time the parent plant dies, its skin bursts and the young, if they may be so called, are liberated, in turn to bud and pass away."

"That seems easy, but what is meant by the product of bacteri, the micrococcus, bacillus and spirillum?"

"The products are the excreta, the excretions. You know that in the vegetable kingdom flowering plants give forth a pure oxygen which is healthful. So bacteria give forth products which are poisonouss. Let me qualify that. It is likely that there are some varieties which are poisonous. Let me qualify that. It is likely that there are same varieties which are innocuous. Now you have the definition of ptomaines, which in brief are products of bacteria."

"And ice cream, that rare delicacy affected by most church festivals and the delight of young people, may contain ptomaines?"

"Yes. A word about that. You see bacteria thrive upon albumen, and albumen is present in milk and is the chief component of gelantine. The latter is found in considerable quantity in the cheaper grades of ice cream. Bacteria are not found in the milk when it comes from the cow; but consider how long a time elapses from that time until it is served to guests in the form of ice cream. Consider that the fluid has been put in cans which have been used and used again, some, perhaps, for years. Remember that bacteria are tenacious of life, and that millions of them may be in the vessel that first held the milk, or in that to which it was transferred, or that in which the cream is placed. Reflect, too, that the extreme of life with these minute organisms is at the most not more than six days, while the duration of life with many is not more than two or three days. Capable of infinite reproduction, and giving off ptomaines in lively quantity, you can readily understand that, planted in a favoring soil like milk or gelatine, where bodily heat is reached, there may very soon be sufficient poison present in the system to superinduce death."

"But surely all ice cream is not poisonous? This is asked for the sake of the millions of lassies who would surrender much of their happiness in this world with the giving up of this compound."

"By no means. Given vessels that are clean of the bacteria and its product, and pure, fresh material, it is as harmless as it is pleasant to the taste."

"You say, though, that it may be present in other foods. Are none exempt from its baleful influences?"

"Recollect that bacteria may be found anywhere. I have said they feed upon albumen, but I will nor deny that varieties may be found which thrive upon oily substances. As far as research has gone, however, butter, say, is safe from it. The presence of ptomaines has been detected in milk used for the artificial feeding of babies; this in cases where the little one suffered from summer complaint. As I said before I took the ice cream as a sort of text because tyrotoxicon has been so positively determined. I repeat that ptomaines are found where ever bacteria have carried on life processes, and foods which incite to activity may certainly contain it whatever those foods may be. Let a bowl of broth be planted with these organisms and in a few days at the most it will be found to be filled with these ptomaines. I have said they thrive under a high degree of temperature, as, for instance, tubercular bacillus does not do well under ninety degrees. Understand, it may live at a much lower, but to thrive it demands the higher temperature."

"And yet the beastly little parasites may be swallowed at any time of the year and in any sort of food?"

"Unquestionably."

"What escape is there, and what happiness is there possible in this world for a fellow who knows not what minute he may take into his stomach that which will bring him to a painful death?" asked the reporter, who by this time felt that he was in the position of the king who would eat of no food that had not been previously tasted by another and felt that even that test was no safeguard for him.

"The lesson of all this is: Eat wholesome, fresh foods and see that they are not offered in unclean vessels or dishes. Let me add that one might swallow very many bacteria without suffering inconvenience. They are so very minute in size and it requires the pormaines from so many of them to cause death. Still the fact remains that many sudden illnesses as well as unexplained deaths should in all probability be assigned to this secret enemy. The scientific investigator speaks cautiously, of course, because so much remains to be found out."

